# Improvement of IFNγ ELISPOT Performance Following Overnight Resting of Frozen PBMC Samples Confirmed Through Rigorous Statistical Analysis

**DOI:** 10.3390/cells4010001

**Published:** 2014-12-24

**Authors:** Radleigh Santos, Alcinette Buying, Nazila Sabri, John Yu, Anthony Gringeri, James Bender, Sylvia Janetzki, Clemencia Pinilla, Valeria A. Judkowski

**Affiliations:** 1 Torrey Pines Institute for Molecular Studies, 11350 SW Village Parkway Port St. Lucie, FL 34987, USA; E-Mail: rsantos@tpims.org; 2 Torrey Pines Institute for Molecular Studies, 3550 General Atomics Court, San Diego, CA 92121, USA; E-Mails: abunying@tpims.org (A.B); nsabri@tpims.org (N.S); 3 Cedars-Sinai, 8700 Beverly Blvd., Los Angeles, CA 90048, USA; E-Mail: John.Yu@cshs.org; 4 ImmunoCellular Therapeutics Ltd., 23622 Calabasas Road, Calabasas, CA 91302, USA; E-Mails: anthony.gringeri@imuc.com (A.G.); james.bender@lionbio.com (J.B.); 5 ZellNet Consulting, 555 North Ave., Fort Lee, NJ 07024, USA; E-Mail: sylvia@zellnet.com

**Keywords:** overnight resting, PBMC, IFNγ ELISPOT, immune monitoring

## Abstract

Immune monitoring of functional responses is a fundamental parameter to establish correlates of protection in clinical trials evaluating vaccines and therapies to boost antigen-specific responses. The IFNγ ELISPOT assay is a well-standardized and validated method for the determination of functional IFNγ-producing T-cells in peripheral blood mononuclear cells (PBMC); however, its performance greatly depends on the quality and integrity of the cryopreserved PBMC. Here, we investigate the effect of overnight (ON) resting of the PBMC on the detection of CD8-restricted peptide-specific responses by IFNγ ELISPOT. The study used PBMC from healthy donors to evaluate the CD8 T-cell response to five pooled or individual HLA-A2 viral peptides. The results were analyzed using a modification of the existing distribution free resampling (DFR) recommended for the analysis of ELISPOT data to ensure the most rigorous possible standard of significance. The results of the study demonstrate that ON resting of PBMC samples prior to IFNγ ELISPOT increases both the magnitude and the statistical significance of the responses. In addition, a comparison of the results with a 13-day preculture of PBMC with the peptides before testing demonstrates that ON resting is sufficient for the efficient evaluation of immune functioning.

## 1. Introduction

Immune monitoring is an essential component of immunological research, translational science and even clinical settings. Many different assays are available that allow the assessment of the immune system for a variety of phenotypes and functions [1]. These assays differ in their sensitivity, specificity, complexity, difficulty of validation and robustness. While some allow the assessment of the phenotypic or structural attributes of specific immune cells (e.g., MHC-peptide multimer staining), others measure their functional features (e.g., ELISPOT) and some both (e.g., intracellular cytokine staining). A key element for the functional evaluation of immune cells is the integrity of the sample. No test can compensate for flaws in the sample integrity. Many important sample preparation steps have been identified that aid in optimal sample preservation, including:

1. The choice of anti-coagulant [2],
2. The time frame between blood draw and PBMC isolation [3,4,5],
3. The storage/shipping temperature of unprocessed blood [6],
4. The effective removal of granulocytes/avoidance of granulocyte contamination [5,7],
5. The optimal cryopreservation with pretested freezing medium that supports sample integrity [8],
6. A careful thawing process [2,9] and
7. The use of pretested media without suppressive or non-specific stimulation features [10].

Two of the major factors for the suppression of functionality are the contamination of the sample with apoptotic cells, especially after cryopreservation [11,12], and the loss of a functional signaling platform in samples obtained from whole blood (e.g., PBMC) [13].

Apoptotic cells can be found to varying degrees in most PBMC samples, with an average of about 5% in thawed PBMC samples obtained from blood processed within eight hours of blood and frozen with pretested freezing media [14]. It has been shown that an increased number of apoptotic cells in a PBMC sample dramatically decreases the functionality of CD4 and CD8 cells as assessed by ELISPOT [11]. Based on a series of experiments in which Smith *et al.* correlated the degree of apoptosis in a sample with its ELISPOT responses against viral epitopes under the same standard operating procedure (SOP), a quality acceptance criterion for PBMC samples evaluated under that SOP was established using their degree of contamination with apoptotic cells (<18%). Further, Lenders showed in a series of experiments that apoptotic cells do not only contaminate the sample and decrease the amount of viable and, hence, potential responder cells, but that they also interfere with antigen processing [12].

In early international proficiency panel studies, laboratories that assessed the amount of apoptotic cells in a thawed sample reported lower viability counts (on average, 4%–6% lower) compared to labs that reported viability based on trypan blue assays (which count apoptotic cells as living cells), but performed better overall, with a higher response detection rate [14]. In addition, these studies also revealed that laboratories that implemented an overnight (ON) resting step had a favorable response detection and better panel performance [14,15]. This held true for repeated panel rounds, and ON resting was included as one of the recommendations of the initial ELISPOT harmonization guidelines, which have been shown to positively affect the ELISPOT performance and decrease the variability in results reported by different labs evaluating the same samples [16,17]. Recently, Kutscher *et al.* demonstrated that ON resting of cryopreserved PBMC leads to lower apoptotic cell and higher dead cell counts in the post-rest sample, illustrating that the resting procedure aids in the removal of apoptotic cells [18].

In the presented study, we investigate the effect of ON resting of PBMC with impaired integrity due to delayed processing and long-term cryopreservation on their recall function to CD8-restricted peptides as measured by IFNγ ELISPOT. Using a modification of the existing distribution free resampling method (DFR) recommended for the statistical analysis of ELISPOT data [19], we apply *p*-value correction across all samples and stimuli, ensuring that the significances presented herein are subject to the most rigorous possible standard and that the resulting analyses have enhanced integrity. The study used five HLA-A2 peptides from the CEF (Cytomegalie, Epstein Barr, and Influenza virus) pool of 32 peptides, and it is denoted as A2-CEF. This pool provided reliable results when comparing the response to the five individual peptides, supporting its use for the immune monitoring of HLA-A2 samples. Lastly, the study includes a comparison of the results obtained from ON resting of cells with results obtained after a 13-day-long *in vitro* stimulation (IVS) prior to testing. The results demonstrate that ON resting is sufficient for rescuing a recall response and its strength, as determined by statistical testing.

## 2. Experimental Section

This section was written in compliance with the Minimal Information about T-cell Assays (MIATA) guidelines [20]. These studies were conducted with established laboratory research protocols following exploratory research principles. Raw data are shown in Supplementary Tables 1S and 2S. The MIANKA (Minimal Information about NK cell Assays) and MIATA sub-modules are shown in Supplementary Table 3S.

### 2.1. Human PBMC Samples

Elutriated apheresis products from four HLA-A*0201 healthy donors (denoted H3, H4, H10 and H12), using citrate phosphate dextrose solution as an anti-coagulant, were obtained from Hemacare Corporation (Van Nuys, California). Apheresis products were received at a temperature of 15 to 20 °C and diluted with phosphate-buffered saline (PBS, Hyclone, Logan, UT) at a 1:1 ratio, aliquoted into 50-mL tubes, spun down (1500 rpm, for 5 min) and suspended into RPMI1640 medium supplemented with 10% fetal bovine serum (FBS; Hyclone, Utah) for counting. The FBS lot was chosen based on optimal cell culture performance. The number of recovered PBMC upon processing ranged from 12–16 × 10^9^ cells per apheresis bag with an average viability of 94%. PBMCs were suspended in serum-free freezing medium (Cryostore10, Biolife Solutions, Bothel, WA) at a density of 1 × 10^8^ cells/mL in 2.0-mL cryovials at 1 mL per vial. Vials were frozen using a Mr. Frosty cryo-freezing container (Nalgene, Thermo Fisher Scientific, Waltham, MA) at −80 °C and transferred to liquid nitrogen within 24 h. The median time from finishing elutriation at Hemacare to the end of cell processing at the Torrey Pines Institute for Molecular Studies (TPIMS) was about 36 h. Apheresis products from donors H3 and H4 were processed at Cedars-Sinai Medical Center, as described above, and shipped ON in vapor nitrogen containers to TPIMS, where the experiments where performed.

### 2.2. Pretest Handling of PBMC

PBMC were thawed in a 37 °C water bath, transferred to 50 U/mL of Benzonase-containing (Millipore, Darmstadt, Germany) culture medium made up of X-Vivo 15 medium (Lonza, Basel, Switzerland), 2 mM l-glutamine), 1% penicillin/streptomycin (all from Hyclone, Waltham, MA) and 10% human AB serum (HS) (GemCell, West Sacramento, CA; pretested for optimal allogeneic expansion of human T-cell clones), washed and resuspended in culture medium without Benzonase. An aliquot of the cells was taken and counted with an automated cell viability counter based on trypan blue inclusion (Cellometer Auto 1000, Nexcelom, Lawrence, MA) prior to the evaluation with IFNγ ELISPOT. Viability in all experiments was confirmed to be from 50%–77% and the cell number recovered between 1.6 and 8.4 × 10^7^ per vial.

For PBMC with a resting period, the final concentration of the cells was adjusted to 2 × 10^6^ cells/mL, and 5 mL were added to 50-mL conical tubes containing no more than 10 × 10^6^ cells. The PBMC were rested ON for 18 or 22 h at 37 °C in a 5% CO_2_-humidified incubator with tube caps slightly loosened for gas exchange. The next day, the rested PBMC were washed twice, counted and resuspended in culture medium before seeding on an ELISPOT plate. The median viability of all of the samples after thawing was 65%, and the median recovery of “live” cells after ON resting for all samples was 67%, ranging between 34% to 100%. The viability and recovery did not vary significantly among the different resting times tested. PBMC that underwent an *in vitro* stimulation (IVS) were handled similarly to the *ex vivo* PBMC without the ON resting. The cells were thawed, treated with Benzonase, washed and counted. The IVS with antigenic peptides was done in a 24-well plate with a total volume of 2 mL per well. Each well contained 4 × 10^6^ PBMC with a final concentration of 1 μg/mL of each peptide and 20 IU/mL of rhIL-2 (R&D Systems, Minneapolis, MN). The plates were incubated at 37 °C in a 5% CO_2_-humidified incubator, and half of the media was changed and replenished with culture medium containing rhIL-2 on Days 4, 6, 8 and 11. On Day 13, the cells were harvested, washed twice, counted and resuspended for assessment by IFNγ ELISPOT. The median viability for all sample cultures upon IVS stimulation was 68%.

### 2.3. ELISPOT Assay

**Figure 1:**
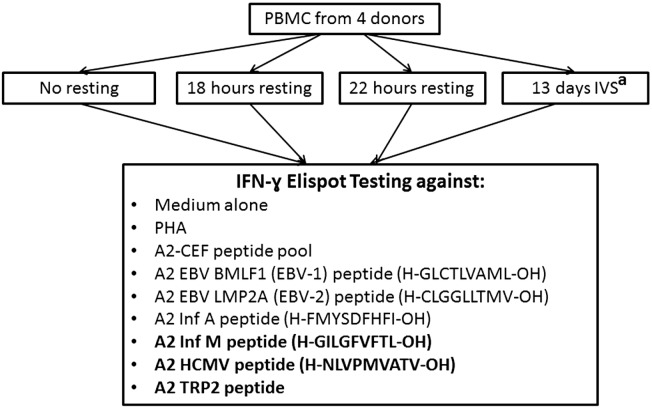
Summary of the experimental conditions used in the study. IVS, *in vitro* stimulation; PHA, phytohemagglutinin; CEF, Cytomegalie, Epstein Barr, and Influenza virus. ^a^ Only the peptides shown on bold were tested in the 13-day IVS.

### 2.4. Data Acquisition

Wells were scanned and counted with the KS ELISPOT Imaging System (Carl Zeiss, Inc., Thornwood, NY) using KS ELISPOT software Version 4.9. The operator had previously participated in the Cancer Immunotherapy Consortium’s ELISPOT Plate Reading Panel, and the established consensus guidelines for plate evaluation were used to evaluate the ELISPOT plates. Specifically, a working set of counting parameters was established by comparing the appearance of negative control wells (cells alone) and antigen-stimulated wells (A2-CEF). The following parameters were fine-tuned to allow the inclusion of typical spots and the exclusion of artifacts: spot size, spot color and color saturation, intensity of staining (contrast), spot shape and degree of fading of color from spot center to its periphery. For each sample and experiment, the applicability of the working set of parameters was tested, and adjustments were made when the conditions required it (e.g., spot crowdedness required the adjustment of the spot separation algorithm; occurrence of many artifacts required the adjustment of spot-defining parameters for that sample, in order to efficiently exclude artifacts from being counted). Counts were always checked for plausibility, and all readings were audited. Representative datasets are provided (Figure 4).

### 2.5. Statistical Analysis

The use of Westfall–Young max-T correction on the difference between the log of the test sample count and the log of the control sample count (referred to as DFR(eq)) or on the difference between the log of the test sample count and twice the log of the background control count (referred to as DFR(2x)), has previously been shown to be the most powerful approach to analyzing single-experiment ELISPOT data while maintaining meaningful significances [19]. However, the goals of this study necessitated direct comparison of multiple experiments, in which multiple-comparison-related reduction of significance remained a problem using the existing DFR framework. We have therefore modified the DFR analysis procedure to carry out the multiple comparisons and to ensure that the significances presented in the study are meaningful.

**Figure 2:**
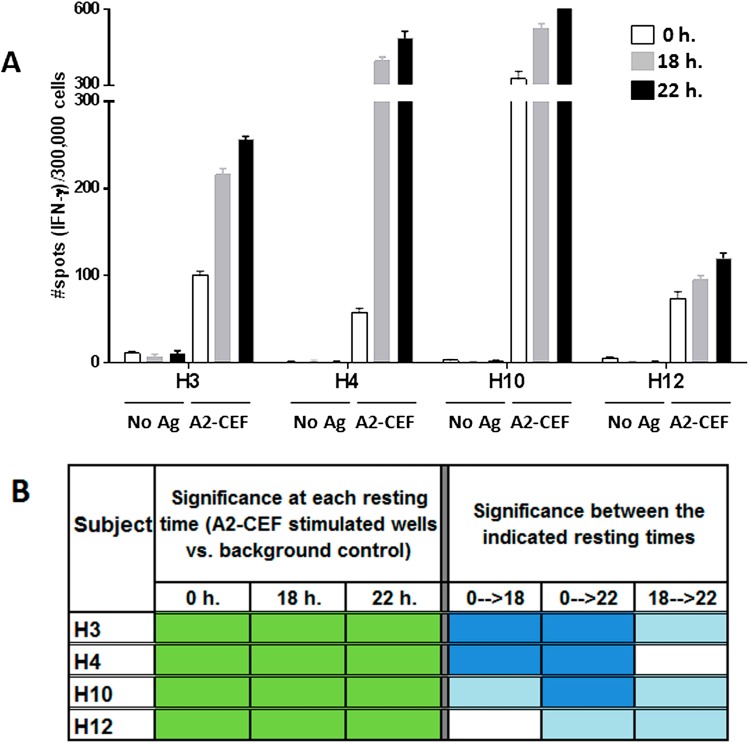
The magnitude of the response to the A2-CEF peptide pool in PBMC samples from normal donors increases with overnight resting. PBMC samples from the indicated donors were thawed and seeded in the presence and absence (no Ag, background control) of the A2-CEF peptide pool after 0 h, 18 h. or 22 h. of resting. (**A**) Results are the average of spots from triplicates wells. (**B**) Statistical significance for the responses to A2-CEF as compare to No Ag at each resting time were determined by modified DFR(2x) (DFR, distribution free resampling) after Westfall–Young max-T correction; p-values <5% are shown in green. The statistical significance of the responses obtained for the three different resting time points was determined by a DFR-like permutation method with Westfall–Young max-T correction; 5% or 10% significances are shown in dark blue or light blue, respectively.

**Figure 3:**
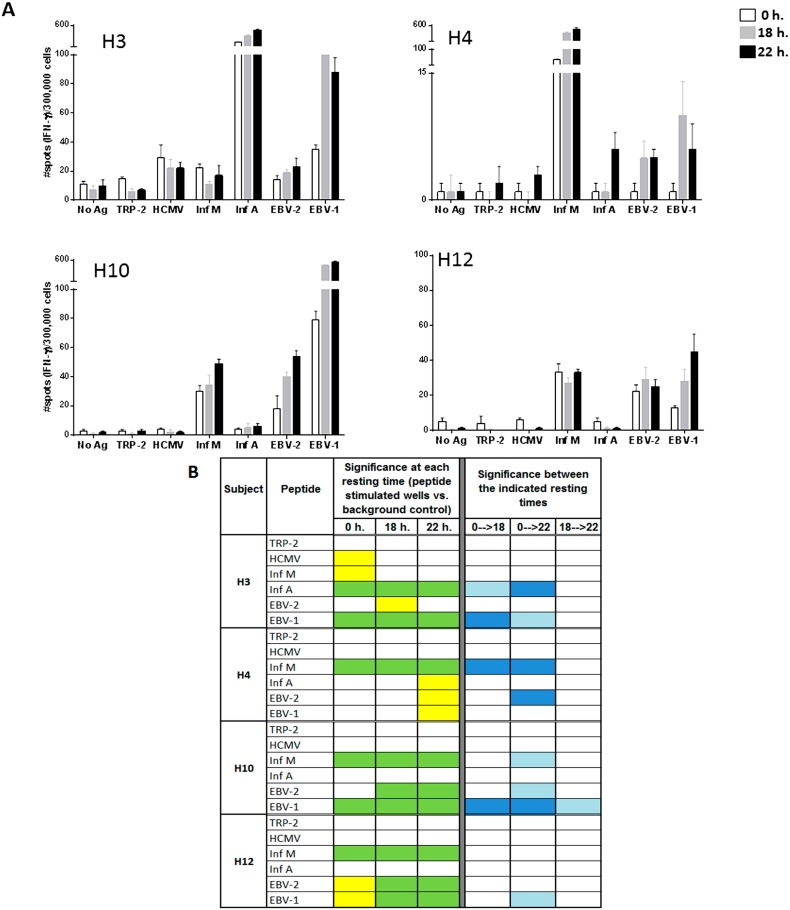
Resting of PBMC samples prior to the evaluation of individual peptides of the A2-CEF pool increases the magnitude of response detected by IFNγ ELISPOT. PBMC samples from the indicated donors where thawed and seeded in the presence or absence (no Ag, background control) of peptide. The peptides included TRP-2 and each of the individual peptides from the A2-CEF pool. PBMCs were not rested (0 h) or rested 18 h or 22 h prior to testing. (**A**) Results are the average of spots from triplicate wells for wells tested with peptide and from six replicates in the absence of peptide. (**B**) The statistical significance for the response at each of the rest times was determined by modified DFR(2x) or DFR(eq) after Westfall–Young max-T correction, and *p*-values <5% are shown in green or yellow, respectively. The statistical significance of the responses obtained for the three different resting time points were determined by a DFR-like permutation method with Westfall-Young max-T correction; 5% or 10% significances are denoted by dark blue or light blue, respectively.

The ELISPOT spot results were analyzed for statistical significance at a given time point by comparing the number of spots for each of the tested peptides (triplicates) with the background controls (no peptide added, six replicates) at that time point. All *ex vivo* data from all experiments (Figure 2B and Figure 3B, Table 1 and Supplementary Tables 1 and 2) had *p*-values simultaneously generated using the non-parametric one-tail Westfall–Young max-T correction as in DFR. Instead of the log-differences used in DFR, however, the test statistics:

(1) $$T=\frac{\bar{X_{T}}-\bar{X_{C}}}{\sigma_{pooled}}$$

and:

(2) $$T=\frac{\bar{X_{T}}-2\bar{X_{C}}}{\sigma_{pooled}}$$

were used, where $\bar{X_{T}}$ is the average count of the test sample replicates, $\bar{X_{C}}$ is the average count of the control sample replicates and:

(3) $$\sigma_{pooled}=\sqrt{\frac{\left(N_{T}-1\right){\sigma_{T}}^{2}+\left(N_{C}-1\right){\sigma_{C}}^{2}}{N_{T}+N_{C}-2}}$$

is the pooled standard deviation, where $N_{T}$ and $N_{C}$ are the sample sizes for the test and control samples (here, three and six) and $\sigma_{T}$ and $\sigma_{C}$ are the test and control sample standard deviations (SD), respectively. To avoid errors associated with artificially small variances, the minimum value allowed for $\sigma_{pooled}$ was $\frac{\sqrt{30}}{10}$ , the maximum standard deviation of an n = 6 binary set. This choice of test statistic, because it is naturally normalized to the order of magnitude of the count, allows for comparison across different levels of activity without strongly active samples skewing the *p*-values, as they would with the log difference. We refer to this method as the modified DFR(eq) when:

(4) $$T=\frac{\bar{X_{T}}-\bar{X_{C}}}{\sigma_{pooled}}$$

was used and the modified DFR(2x) when:

(5) $$T=\frac{\bar{X_{T}}-2\bar{X_{C}}}{\sigma_{pooled}}$$

was used. We refer to a result as strongly significant if the DFR(2x) *p*-value of that result is less than 0.05 and moderately significant if it is not strongly significant, but the DFR(eq) *p*-value is less than 0.05. All *p*-values for IVS results (Table 1) were also generated simultaneously in the same manner and with the same nomenclature for significance and the same sample sizes.

The statistical significance of the magnitude of responses between time points for each peptide was determined by comparing the normalized triplicate values for each peptide. The normalization at each time was done by subtracting the average of the six background replicates of the background controls to each of the triplicates of the peptide responses. *p*-values for comparisons between time points (Figure 2B and Figure 3B) were then generated simultaneously using Westfall–Young max-T correction as in DFR, but again with a modified test statistic:

(6) $$T=\frac{\left(\bar{X_{T1}}-\bar{X_{C1}}\right)-\left(\bar{X_{T2}}-\bar{X_{C2}}\right)}{\sigma_{pooled}},$$

written here to include the aforementioned normalization. Here,

(7) $$\sigma_{pooled}=\sqrt{\frac{\left(N_{T1}-1\right){\sigma_{T1}}^{2}+\left(N_{T2}-1\right){\sigma_{T2}}^{2}}{N_{T1}+N_{T2}-2}},$$

with:

(8) $$N_{T1}=N_{T3}=3$$

Strongly significant refers to a result with a *p*-value less than 0.05 and moderately significant to a *p*-value less than 0.10.

## 3. Results and Discussion

### 3.1. Overnight Resting of PBMC Samples Increases the Magnitude of the Response to the A2-CEF Pool

**Figure 4:**
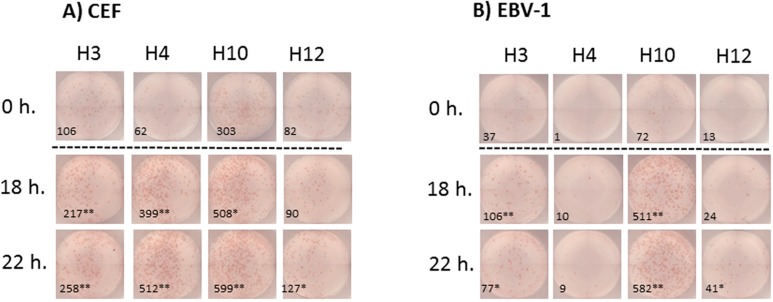
Representative images of the IFNγ ELISPOT performed with previously frozen PBMC. PBMC samples from donors H3, H4, H10 and H12 were thawed and stimulated (**A**) with the A2-CEF pool or (**B**) with EBV-1. PBMCs were not rested (0 h) or rested for 18 h or 22 h prior to testing. Each picture is a representative of triplicate wells. Responses that were significantly different from 0 h were determined by a DFR-like permutation method with Westfall–Young max-T correction; 5% or 10% significances are denoted by (**) or (*), respectively.

To evaluate the impact of ON resting of PBMC samples on the responses measured by IFNγ ELISPOT testing, PBMCs from four different HLA-A*0201 individuals were tested for their reactivity to a pool of five HLA-A2.1-restricted viral peptides from influenza (Inf) virus, EBV and HCMV (A2-CEF; an A2-condensed version of the CEF pool, known as a reliable control for detecting CD8 memory T-cell responses in PBMCs) [21]). Different vials of PBMC samples were non-rested (0 h. resting) or rested for 18 h or 22 h before testing. Responses from all donor PBMC samples to the A2-CEF pool at all three time points (0, 18 and 22 h) were positive and strongly statistically significant when compared with background control wells (PBMC alone) (see Figure 2A, Figure 2B, left, and the representative images of the ELISPOT results in Figure 4A). Although qualitative detection, as a matter of statistical significance, would not change based on whether the cells were rested (the *p*-values for all of the subjects at the three time points are <5%, shown in green on the left of Figure 2B), it is evident that the magnitude of the responses drastically increased after resting (Figure 2A and Supplementary Table 1). In particular, the magnitude of the response to the A2-CEF pool was statistically different between 0 h and 22 h. for all PBMC donor samples and statistically different between 0 h and 18 h for 3 PBMC donor samples (H12 being the exception), with the spot counts as measured at 22 h being higher (Figure 2A, right of Figure 2B and Supplementary Table 1). Furthermore, for three out of the four donors, PBMC responses were even significantly different between 18 h and 22 h (H4 being the exception).

### 3.2. Overnight Resting of PBMC Samples Increases the Magnitude and Statistical Significance of Responses to Individual Peptides of the A2-CEF Pool

To more effectively measure the effect of ON resting, each of the A2-CEF peptides included in the A2-CEF pool was tested individually (Figure 3 and representative images for EBV-1 in Figure 4B). TRP-2, a tumor associated antigen, was also included as a control. As summarized in Figure 3B, after ON resting, donors had not only more strong statistically significant responses to peptides, but also developed new moderate significant responses (left). Further, the magnitude of response upon resting increased significantly in multiple cases. The raw spot counts for each of the triplicate wells used for the evaluation of the peptides and for each of the six replicates used for background control are shown in Supplementary Table 1.

Looking at specific examples, the response to EBV-2 by H10 is statistically significant only when cells are rested, and the statistical significances for EBV-2 and EBV-1 by H12 changes from moderate (yellow) to strong (green) when cells are rested for at least 18 h. The responses to Inf A, EBV-2 and EBV-1 by H4 are significant only after resting 22 h, albeit only moderately so (Figure 3B, left). Further, the magnitude of the response was found to be strongly statistically significant between no resting and either 18 h or 22 h of resting for Inf A and EBV-1 by H3, Inf M and EBV-2 by H4 and EBV-1 by H10 (Figure 3B, right). Out of the total of 20 responses to the individual viral peptides evaluated in the four donors (4 donors × 5 single viral peptides), four responses (20%) were significantly different at 18 h compared to no resting and eight (40%) were significantly different at 22 h compared with no-resting. Furthermore, the magnitude of the response was only statistically different between 18 and 22 h for EBV-1 by donor H10. This analysis suggests that 22 h of resting could be even more advantageous than 18 h, but an 18-h resting period appears to be a sufficient resting timeframe for evaluating the T-cell responses to individual peptides. Of interest is the fact that the impact of resting the samples has been less remarkable in donor H12, whose samples have been stored in liquid nitrogen for a shorter time than the other three donors tested. Future studies including more samples and more variable storage periods could be considered.

As can be observed in Figure 3B, many responses remained undetectable, even when resting the cells, such as the response to HCMV by H4. Indeed, out of the 14 responses that were negative without resting among all four donors, nine remained negative and five became positive after 18 or 22 h. of resting (Figure 3B). The only response that was detected without resting and not detected with resting was to HCMV and Inf M by donor H3; however, they were only moderately statistically significant, and this lack of detection after resting was not reproducible in an earlier experiment. In that experiment, moderate (Inf M) or strong (HCMV) significance resulted after resting 22 h., as well; see Supplementary Table 2. Lastly, no responses to TRP-2 were detected by any of the subjects, at any time point. This finding was not surprising, since TRP-2 is a tumor-associated antigen and the PBMCs used in this study were obtained from healthy donors. These results emphasize the specificity of resting effects on antigen-specific responses and demonstrate that resting does not cause a non-specific increase of spot counts or induce false positives.

### 3.3. The Quality of the Responses Is Not Improved by *In Vitro* Stimulation

To assess whether IVS of PBMC with the viral peptides would improve the response detection, PBMCs of all four donors were stimulated for 13 days with two viral peptides and the tumor antigen TRP-2 and tested in ELISPOT. The results are shown in Table 1. Significances were calculated as described in the Experimental Section, but for a clearer demonstration of the magnitude of effect the data presented in the table, there is as an average fold increase (FI) over the background. FI values higher than 50 are derived either from peptide-stimulated wells that had counts 50-times higher than the background or too many spots to count (higher than 2000). As can be observed, all strongly significant IVS results were also at least moderately significant in *ex vivo* testing after resting. Although H12 stimulated with HCMV changes from not significant to moderately significant after IVS, the background reactivity observed in the IVS without any peptide (negative control) puts the overall response detection to peptides into question. Indeed, a similar observation was made for the response by H10 to the negative control TRP-2 peptide, which is moderately statistically significant for the IVS performed without peptide, but negative for the IVS with TRP-2 peptide. Overall, these data suggest that IVS does not provide clear advantages for detecting memory responses to viral peptide.

**Table 1:** The comparison of IFNγ ELISPOT performance using *ex vivo* *vs.* IVS-PBMC samples shows that IVS stimulation prior to testing does not provide clear advantages for the determination of peptide immunogenicity. PBMC samples from the indicated donors were either thawed and rested for 22 h. (*ex vivo*) or thawed and stimulated with a pool of TRP-2, HCMV and influenza M peptides during 13 days (IVS-peptide stimulated) or thawed and left unstimulated for 13 days ( IVS-no peptide) prior to testing with each of the indicated individual peptides. The table shows the fold increase calculated as follows: AVG (average) of spots from triplicate wells tested with peptide/AVG of spots from six replicate background wells (no peptide). The statistical significance for the differences between the number of spots in the presence of peptide and background was determined by modified DFR(2x) or DFR(eq) after Westfall–Young max-T correction; *p*-value <5% responses are shown in green or yellow formatting, respectively.

	*Ex Vivo*				IVS-Peptide Stimulated				IVS-No Peptide			
Donor/Peptide	H3	H4	H10	H12	H3	H4	H10	H12	H3	H4	H10	H12
**TRP-2**	1.9	0.5	0.6	0.5	0.6	0.7	1.2	1.3	1.6	1.5	1.3	1.1
**HCMV**	5.3	1.0	0.9	0.2	>50	0.7	1.0	1.7	2.7	0.7	1.1	1.3
**Inf M**	4.4	>50	11	19	11	>50	>50	>50	1.7	33	1.4	1.2

### 3.4. Discussion

In this study, a condensed version of the CEF peptide pool, containing only five A2-restricted peptides (A2-CEF), was used. Clearly, the strong significance level of A2-CEF responses observed in these study indicate that the A2-CEF peptide pool can be an effective control for CD8+ memory responses in HLA-A2 subjects and may be of interest in light of cost-preserving measures. In immune monitoring efforts that include large panels of clinical samples with the need for confirmatory assay repeats, this can represent a significant cost savings.

Equally apparent in the data is the advantage of resting the cells. Figure 2A and Table S1 clearly show that the number of spots in wells tested with peptide is higher in most cases after resting. In addition to the response to EBV-2 by H10 being undetected without resting, but detected with strong significance after resting 18 h, and the responses to Inf A, EBV-2 and EBV-1 by H4 being moderately detected only after 22 h resting, there was also an improvement in the significance of the detected stimulation from moderate to strongly significant in the responses to EBV-2 and EBV-1 by H12. Indeed, of the nine strongly significant results for individual peptides after resting 18 h, only two thirds were strongly significant without resting. The advantages to increasing the level of detected significance should not be underestimated, because, in general, moderately statistically significant results are less likely to reproduce than strongly significant results; as previously reported [19], DFR(eq) has a false positive rate of 10.7%, over five-times higher than that of DFR(2x) (2.0%). Direct comparisons between spot counts associated with not resting *versus* resting also support this conclusion, with eight subject/stimulus combinations showing at least moderate significance between no resting and either (or both) 18 and 22 h of resting. Note that these significances do not always track with increases in significance overall. If the sample is already significant without resting, for example, then the significance between resting times will not be evident in the significance at each resting time. Conversely, because significances between resting times were the results of comparisons between two samples of a size of three, rather than three *versus* six background samples, as was the case of measuring the significance at each resting time, a change in significance after resting does not necessarily imply significance between those two measurements.

Overall, moderately significant responses (yellow) were found in wells in which the number of spots was low (less than approximately 35). An excellent example of one way in which resting is advantageous to such a moderately significant sample is shown through analysis of the response of donor H12 to EBV2. The response to the peptide did not result in a particularly high spot count at 0 h., and the average spot count did not increase after resting; rather, there was a decrease in the number of background spots. Reduction in the number of spots for the background wells after resting for 18 and 22 h contributed to the increase of the statistical significance of the difference between background wells and peptide response (Supplementary Table 1), even though the unrested background number of spots was not unreasonably high. This shows that the background can be “cleaned up” by resting. Another observed example of such “cleaning up” was the removal of testing artifacts after resting (Figure 1S). Although this finding was observed in only one out of the four donors included in this study, it suggests that when analyzing a large number of donors, a nontrivial percentage of the samples may present artifacts in the background and, thus, will benefit from ON resting for the determination of peptide-specific T-cell responses.

Both of these observations, the increase of spot counts and the decrease of artifacts, underline the applicability of the previously described benefits of ON resting. Small artifacts in ELISPOT are most commonly caused by dying cells during the assay incubation time. As Kutscher *et al.* convincingly showed, cells die during prolonged resting periods [18]; hence, less apoptotic cells are added to the actual assay, giving less cause for artifacts. Indeed, the percent recovery determined in the presented study also supports that observation. The median recovery of the seeded “live” cells for all samples after resting was 67%. Although, in this study, apoptotic markers were not used to confirm that these lost cells were undergoing apoptosis, our data shows that resting the cells is beneficial for reliable determination of peptide-specific T-cell responses. Future studies comparing the performance of freshly isolated PBMC with rested cryopreserved samples could be performed in order to determine which of the tested resting conditions more accurately resemble the freshly-isolated PBMC performance. In addition, the fact that cells were cryopreserved at a high concentration (100 × 10^6^ cells/vial) could have contributed to the effect of overnight resting; however, the results presented here clearly show that samples that are potentially impaired due to processing steps can highly benefit from the effects of overnight resting prior to testing. ELISPOT results using rested PBMC samples derived from clinical studies that have been cryopreserved at a concentration of 5 to 20 × 10^6^ cells/vial have shown, in general, low background responses and variance (manuscript in preparation) [22].

It should also be noted that the improvements associated with ON resting are not simply an alternative to using more cells, even if it is practical to do so (*i.e.*, cell scarcity is not an issue). A doubling of all values in this study would lead to exactly the same significances, since the factor of two would cancel in the numerator and denominator of the test statistic. A doubling of the number of replicates would not offer substantial benefit either, as it would only improve non-significant samples with high variance. Resulting from the multiple aforementioned biological processes that occur during ON resting, the improvement in detectability of responsive cells presented here as a result of ON resting is far more universal in its benefit, especially since non-responders continue to be non-significant rather than presenting as false positives.

Furthermore, using polychromatic flow experiments, Kutscher *et al.* demonstrated that the quantity, as well as the quality of T-cells responding to viral antigens changes after resting. As the mono-functional T-cell fraction decreased upon ON resting, the fraction of multi-functional T-cells, as well as their antigen sensitivity increased. Importantly, tetramer assessment revealed that the actual number of TCR-specific T-cells does not change during the resting period. This fact is important for obtaining reliable estimates of true precursor frequencies of antigen-specific T-cells, even after ON resting.

An enlightening insight into the mechanism related to the benefits (as the increase in responses, as observed in the study presented here) of resting cells before functionally assessing them is given by Roemer *et al.* [13]. Their work is based on the acknowledgement that the cells typically assessed in most studies consist of circulating cells (PBMC), which contain less than 1% of the body’s T-cells. It had already been shown in the murine model that T-cells entering the circulation lose their primed stage [23]. Roemer shows that the expression of phosphotyrosine, a key player of the signal complex assembly, is low in circulating T-cells, but is regained after high density culture, correlating with a recovery of CD4 functionality. The cellular interactions during the high density resting period were necessary for functional maturation, which include the provision of weak TCR signals from HLA scanning [24], as well as the upregulation of the sensitivity to TCR signals [25]. Similar effects of resting periods were recently presented for CD8+ cells, postulating that high density preculture of PBMC resets cells to a tissue-like state with a proper functioning signaling platform [26].

Interestingly, the data presented here show that *in vitro* culture of PBMCs for 13 days results in a similar qualitative detection of antigen-specific T-cells at 22 h. There was only one case in which IVS led to the detection of a significant response that resulted in being negative when tested *ex vivo* (H12 with HCMV, Supplementary Table 2).

The ELISPOT results associated with this study comprised a total of 164 separate comparisons between stimulated and background control wells, over all subjects and stimuli. Studies that analyze large datasets to determine a significant change in response, but that use arbitrary criteria, such as doubling of spot numbers [27] rather than *p*-values and statistical significances *versus* background, lack analytical rigor. In particular, these methods are incapable of giving *p*-values and correcting for multiple comparisons and, hence, cannot quantify the Type I error (*i.e.*, the theoretical false positive rate) of the study. The importance of accounting for multiple comparisons in the determination of statistical significance has been previously highlighted [19,28]. Also highlighted in these publications were the difficulties associated with differing orders of magnitude of response; the standard DFR framework, which uses difference in the log of the average spot number as its test statistic, corrects within a given stimulus, but does not correct between stimuli, because the magnitude of two stimulus responses can disrupt the ability for a permutation method, like Westfall–Young correction, to properly account for sample randomness. Herein, we have attempted to overcome this issue via using an alternative test statistic, namely the standardized difference between the sample and control means. It should be emphasized that this statistic was the only change to the existing DFR framework of generating *p*-values using Westfall–Young correction; although this statistic is used in the Student’s *t*-test, the Student’s *t*-test was found previously to be suboptimal for these analyses [19], likely because of the failure of the ELISPOT data to be normally distributed. When this statistic is used in the context of Westfall–Young correction, however, it was quite effective at generating adjusted *p*-values across stimuli; the standardized difference between the sample and control means is capable of making comparable data with different orders of magnitude by normalizing the count difference by the standard deviation, placing all data on the same scale (*i.e.*, the number of standard deviations above the control). Thus, in this study, all *p*-values were simultaneously corrected across all experiments, making stronger any findings of significance in the process. In particular, DFR(2x) would have indicated 87 total significant results; modified DFR(2x) indicated only 72, with 10 becoming only moderately significant and five losing significance altogether; DFR(eq) indicated 14 significant results in addition to the above, and 11 of them lost significance in modified DFR(eq). *p*-value corrections of IVS results were more similar to DFR, with five DFR(eq) significant values becoming non-significant and no change in significance in those significant in the DFR(2x) analysis. In general, decreasing the false positive rate can potentially increase the false negative rate, but by making the significance results in this study extremely unlikely to be false positives associated with multiple comparisons, and the results are thus more accurately representative of a true reflection of the pre- and post-resting detection ability in general future contexts.

Previous studies have demonstrated the benefits of ON resting [14,15,29]. Furthermore, two large HIV networks, the HVTN (HIV Vaccine Trials Network) and the IAVI (International Aids Vaccine Initiative), included the ON resting into their SOP [30,31]. However, it should be noted that a recent study [27] has presented an analysis of a seemingly similar experimental set-up without coming to this conclusion. In addition to having methodological differences, such as sample condition, freezing and ON resting methodologies, and differing cut-off criteria, Kuerten *et al.* approached the analysis and interpretation of the data in a manner that is not able to accurately reflect the benefits of ON resting. In particular, by applying a single statistical test to all samples in a given response category, the authors limit themselves to determining whether ON resting causes an overall increase in spot count for all samples. However, stating that the overall effect of ON resting is not significant because all samples do not increase in spot count essentially penalizes samples for not improving after resting. For this to be a valid means of analysis, all samples would have to be expected to yield a positive response; this assumption is not true in the case of the antigens used in the present study and is certainly false in the general research and immunomonitoring setting. Since, in general, there will be samples containing no cells with specific reactivity for some of the antigens tested (*i.e.*, there will be truly negative responders), one needs to specifically focus on the ability of resting the cells to improve the detection of the samples that do contain cells specific for the antigen tested (*i.e.*, are truly positive responders). Indeed, to avoid false positives, it is a benefit if ON resting does not make true negative responses significant. The fact that ON resting does not increase non-specific reactivity has also been previously reported [29]. It is important to note that this distinction in behavior between responders and non-responders can also be observed in the results as presented in Kuerten *et al.*; a greater overall increase in stronger responders (*i.e.*, samples that are most likely to have responsive cells) was observed as compared to weak responders (*i.e.*, samples that may be comprised of truly negative responding cells), implying that the results of these studies may agree and that the difference is a matter of analysis and interpretation of the data. Our analysis demonstrates the improvement in response detection in the most rigorous and non-assumptive statistical way possible, through the generation of distribution-free, multiple comparison-corrected *p*-values, and not by simply grouping possibly disparate responder-types based on unrested response; this strict statistical analysis revealed the clear advantage of ON resting.

Importantly, our observed results fit into recently published observations addressing the benefits and outcomes of extended resting of thawed PBMC on their functionality [13,18,32], as demonstrated with different experimental approaches. ELISPOT is one of the most commonly used tests in the immune monitoring setting. Most studies use frozen PBMC for batch testing. Freezing and thawing procedures can induce an increased rate of apoptosis, leading to a dilution of the test population with non-responsive cells, which may further impact the antigen processing. Further, there is only limited feasibility of obtaining cells from relevant tissue, e.g., tumor-infiltrating T-cells (TILs), exist [33]. If such samples are available, they can only be obtained in small samples sizes, and repeated availability (as for numerous time points) is close to impossible. The resting approach appears to offer a partial solution to the dilemma in that it can provide conditions that aid in resetting T-cells to a tissue-like state with an improved responsiveness to the antigens of interest. Such improved responsiveness in samples that are relatively easy to obtain (e.g., PBMCs) is essential for reliable immune monitoring in translational research and clinical trials, in order to guide the development of biomarkers and new immunotherapies [16].

In summary, the results presented here strongly support the implementation of an ON resting step of previously-frozen PBMC samples for the detection of peptide-specific responses by ELISPOT testing, a step that has already been identified as a critical protocol variable in ELISPOT and, hence, has been incorporated in ELISPOT harmonization guidelines [14,15,30]. One of the reasons for a slow adaptation rate of this recommendation has been the fear of losing too many cells. However, a possible lower recovery of cells after resting has to be accepted, since cells lost were likely undergoing apoptosis and, hence, would have not responded in the assay. As a matter of fact, the cells recovered contain less apoptotic cells, hence posing a lower risk for impaired antigen processing, and contain cells with a recovered signaling platform for efficient immune functioning. Although viral antigens were used in the presented study, these finding should be easily applicable to samples from clinical trial subjects, especially in light of the questionable sample functionality, due to the often apparent difficulties in obtaining PBMCs in a timely manner. The results presented clearly demonstrate the positive influence of extended resting on the quality of the antigen-specific response detection and its statistical significance.

## Supplementary Materials

Supplementary materials can be accessed at: http://www.mdpi.com/2073-4409/4/1/1/s1.

## References

[1] Janetzki S., Romero P., Roederer M., Bolton D., Jandus C., Morrow W.J.W., Sheikh N.A., Schmidt C.S., Davies D.H. (2012). Immune Monitoring Design within the Developmental Pipeline for an Immunotherapeutic or Preventive Vaccine. Vaccinology: Principles and Practice.

[2] Mallone R., Mannering S.I., Brooks-Worrell B.M., Durinovic-Bello I., Cilio C.M., Wong F.S., Schloot N.C. (2011). Isolation and preservation of peripheral blood mononuclear cells for analysis of islet antigen-reactive cell responses: Position statement of the T-cell workshop committee of the immunology of t diabetes society. Clin. Exp. Immunol..

[3] Bull M., Lee D., Stucky J., Chiu Y.L., Rubin A., Horton H., McElrath M.J. (2007). Defining blood processing parameters for optimal detection of cryopreserved antigen-specific responses for HIV vaccine trials. J. Immunol. Methods.

[4] Kierstead L.S., Dubey S., Meyer B., Tobery T.W., Mogg R., Fernandez V.R., Long R., Guan L., Gaunt C., Collins K. (2007). Enhanced rates and magnitude of immune responses detected against an hiv vaccine: Effect of using an optimized process for isolating pbmc. AIDS Res. Hum. Retroviruses.

[5] Afonso G., Scotto M., Renand A., Arvastsson J., Vassilieff D., Cilio C.M., Mallone R. (2010). Critical parameters in blood processing for t-cell assays: Validation on ELISPOT and tetramer platforms. J. Immunol. Methods.

[6] Olson W.C., Smolkin M.E., Farris E.M., Fink R.J., Czarkowski A.R., Fink J.H., Chianese-Bullock K.A., Slingluff C.L. (2011). Shipping blood to a central laboratory in multicenter clinical trials: Effect of ambient temperature on specimen temperature, and effects of temperature on mononuclear cell yield, viability and immunologic function. J. Translat. Med..

[7] McKenna K.C., Beatty K.M., Vicetti Miguel R., Bilonick R.A. (2009). Delayed processing of blood increases the frequency of activated CD11b+ CD15+ granulocytes which inhibit T-cell function. J. Immunol. Methods.

[8] Filbert H., Attig S., Bidmon N., Renard B.Y., Janetzki S., Sahin U., Welters M.J., Ottensmeier C., van der Burg S.H., Gouttefangeas C. (2013). Serum-free freezing media support high cell quality and excellent ELISPOT assay performance across a wide variety of different assay protocols. Cancer Immunol. Immunother..

[9] Smith J.G., Liu X., Kaufhold R.M., Clair J., Caulfield M.J. (2001). Development and validation of a gamma interferon ELISPOT assay for quantitation of cellular immune responses to varicella-zoster virus. Clin. Diagnost. Lab. Immunol..

[10] Janetzki S., Price L., Britten C.M., van der Burg S.H., Caterini J., Currier J.R., Ferrari G., Gouttefangeas C., Hayes P., Kaempgen E. (2010). Performance of serum-supplemented and serum-free media in ifngamma ELISPOT assays for human T-cells. Cancer Immunol. Immunother..

[11] Smith J.G., Joseph H.R., Green T., Field J.A., Wooters M., Kaufhold R.M., Antonello J., Caulfield M.J. (2007). Establishing acceptance criteria for cell-mediated-immunity assays using frozen peripheral blood mononuclear cells stored under optimal and suboptimal conditions. Clin. Vaccine Immunol..

[12] Lenders K., Ogunjimi B., Beutels P., Hens N., van Damme P., Berneman Z.N., van Tendeloo V.F., Smits E.L. (2010). The effect of apoptotic cells on virus-specific immune responses detected using IFNγ ELISPOT. J. Immunol. Methods.

[13] Romer P.S., Berr S., Avota E., Na S.Y., Battaglia M., ten Berge I., Einsele H., Hunig T. (2011). Preculture of pbmcs at high cell density increases sensitivity of T-cell responses, revealing cytokine release by CD28 superagonist TGN1412. Blood.

[14] Janetzki S., Panageas K.S., Ben-Porat L., Boyer J., Britten C.M., Clay T.M., Kalos M., Maecker H.T., Romero P., Yuan J. (2008). Results and harmonization guidelines from two large-scale international ELISPOT proficiency panels conducted by the cancer vaccine consortium (cvc/svi). Cancer Immunol. Immunother..

[15] Britten C.M., Gouttefangeas C., Welters M.J., Pawelec G., Koch S., Ottensmeier C., Mander A., Walter S., Paschen A., Muller-Berghaus J. (2008). The cimt-monitoring panel: A two-step approach to harmonize the enumeration of antigen-specific CD8+ t lymphocytes by structural and functional assays. Cancer Immunol. Immunother..

[16] Van der Burg S.H., Kalos M., Gouttefangeas C., Janetzki S., Ottensmeier C., Welters M.J., Romero P., Britten C.M., Hoos A. (2011). Harmonization of immune biomarker assays for clinical studies. Sci. Transl. Med..

[17] Janetzki S., Britten C.M. (2012). The impact of harmonization on ELISPOT assay performance. Methods Mol. Biol..

[18] Kutscher S., Dembek C.J., Deckert S., Russo C., Korber N., Bogner J.R., Geisler F., Umgelter A., Neuenhahn M., Albrecht J. (2013). Overnight resting of pbmc changes functional signatures of antigen specific T-cell responses: Impact for immune monitoring within clinical trials. PLoS One.

[19] Moodie Z., Price L., Gouttefangeas C., Mander A., Janetzki S., Lower M., Welters M.J., Ottensmeier C., van der Burg S.H., Britten C.M. (2010). Response definition criteria for ELISPOT assays revisited. Cancer Immunol. Immunother..

[20] Britten C.M., Janetzki S., Butterfield L.H., Ferrari G., Gouttefangeas C., Huber C., Kalos M., Levitsky H.I., Maecker H.T., Melief C.J. (2012). T-cell assays and miata: The essential minimum for maximum impact. Immunity.

[21] Currier J.R., Kuta E.G., Turk E., Earhart L.B., Loomis-Price L., Janetzki S., Ferrari G., Birx D.L., Cox J.H. (2002). A panel of MHC class I restricted viral peptides for use as a quality control for vaccine trial ELISPOT assays. J. Immunol. Methods.

[22] Santos R., Buying A., Sabri N., Appel J., Yu J., Gringeri A., Bender J., Janetzki S., Pinilla C., Judkowski V. (2014).

[23] Stefanova I., Dorfman J.R., Germain R.N. (2002). Self-recognition promotes the foreign antigen sensitivity of naive T lymphocytes. Nature.

[24] Garcia K.C., Adams J.J., Feng D., Ely L.K. (2009). The molecular basis of tcr germline bias for mhc is surprisingly simple. Nat. Immunol..

[25] Randriamampita C., Boulla G., Revy P., Lemaitre F., Trautmann A. (2003). T-cell adhesion lowers the threshold for antigen detection. Eur. J. Immunol..

[26] Fischer J., Hackenberg S., Grigoleit G.U., Stevanovic S., Huenig T. Resetting circulating CD8 T-cells to a tissue-like status permits sensitive detection of their responses to virus- and tumor-associated antigens. Proceedings of the 12th Annual Meeting of the Association for Immunotherapy of Cancer.

[27] Kuerten S., Batoulis H., Recks M.S., Karacsony E., Zhang W., Subbramanian R.A., Lehmann P.V. (2012). Resting of Cryopreserved PBMC Does Not Generally Benefit the Performance of Antigen-Specific T-cell ELISPOT Assays. Cells.

[28] Moodie Z., Huang Y., Gu L., Hural J., Self S.G. (2006). Statistical positivity criteria for the analysis of ELISPOT assay data in HIV-1 vaccine trials. J. Immunol. Methods.

[29] Malyguine A., Strobl S.L., Shafer-Weaver K.A., Ulderich T., Troke A., Baseler M., Kwak L.W., Neelapu S.S. (2004). A modified human ELISPOT assay to detect specific responses to primary tumor cell targets. J. Translat. Med..

[30] Gill D.K., Huang Y., Levine G.L., Sambor A., Carter D.K., Sato A., Kopycinski J., Hayes P., Hahn B., Birungi J. (2010). Equivalence of ELISPOT assays demonstrated between major HIV network laboratories. PLoS One..

[31] Gorse G.J., Baden L.R., Wecker M., Newman M.J., Ferrari G., Weinhold K.J., Livingston B.D., Villafana T.L., Li H., Noonan E., Russell N.D. (2008). Safety and immunogenicity of cytotoxic T-lymphocyte poly-epitope, DNA plasmid (EP HIV-1090) vaccine in healthy, human immunodeficiency virus type 1 (HIV-1)-uninfected adults. Vaccine.

[32] Chudley L., McCann K.J., Coleman A., Cazaly A.M., Bidmon N., Britten C.M., van der Burg S.H., Gouttefangeas C., Jandus C., Laske K. (2014). Harmonisation of short-term *in vitro* culture for the expansion of antigen-specific CD8 T-cells with detection by ELISPOT and hla-multimer staining. Cancer Immunol. Immunother..

[33] Gajewski T.F., Schreiber H., Fu Y.X. (2013). Innate and adaptive immune cells in the tumor microenvironment. Nat. Immunol..

