# Correction: Santos, R.S., *et al.* Improvement of IFNγ ELISPOT Performance Following Overnight Resting of Frozen PBMC Samples Confirmed Through Rigorous Statistical Analysis. *Cells* 2015, *4*, 1-18

**DOI:** 10.3390/cells4020133

**Published:** 2015-04-21

**Authors:** Radleigh Santos, Alcinette Buying, Nazila Sabri, John Yu, Anthony Gringeri, James Bender, Sylvia Janetzki, Clemencia Pinilla, Valeria A. Judkowski

**Affiliations:** 1 Torrey Pines Institute for Molecular Studies, 11350 SW Village Parkway Port St. Lucie, FL 34987, USA; E-Mail: rsantos@tpims.org; 2 Torrey Pines Institute for Molecular Studies, 3550 General Atomics Court, San Diego, CA 92121, USA; E-Mails: abunying@tpims.org (A.B); nsabri@tpims.org (N.S); 3 Cedars-Sinai, 8700 Beverly Blvd., Los Angeles, CA 90048, USA; E-Mail: John.Yu@cshs.org; 4 ImmunoCellular Therapeutics Ltd., 23622 Calabasas Road, Calabasas, CA 91302, USA; E-Mails: anthony.gringeri@imuc.com (A.G.); james.bender@lionbio.com (J.B.); 5 ZellNet Consulting, 555 North Ave., Fort Lee, NJ 07024, USA; E-Mail: sylvia@zellnet.com

The authors wish to make the following corrections to this paper [1]:

In Section 2.3 ELISPOT Assay, on page 4, the following text is missing: “PBMC samples from each donor were tested after three different resting time points (0, 18, 22 h.) for their reactivity to a pool of 5 HLA-A2.1-restricted viral peptides from influenza (Inf) virus, EBV and HCMV (A2-CEF) or to each of the A2-CEF peptides individually. TRP-2, a tumor-associated antigen, was also included as a control. The A2-CEF peptide pool or each of the peptides was tested in triplicate. Background controls (no peptide added) were tested by six replicates. See the workflow overview in Figure 1. PVDF 96-well plates (MSIPS4510, Millipore, Darmstadt, Germany) were pre-wetted with 15 μL per well of 70% ethanol and shaken with a plate shaker for 15 seconds. Each plate was then washed three times with PBS without Ca/Mg (Hyclone, Waltham, Massachusetts). Afterwards, the plates were coated with anti-IFNγ antibody (Clone 1-D1k, Mabtech, Cincinnati, OH) at 100 μL per well at a final concentration of 10 μg/mL and wrapped in aluminum foil for ON storage at 4 °C. The following day, the coating antibody was removed by washing with 200 μL PBS per well. Plates were blocked with 150 μL/well culture medium, for at least one hour and up to 3 h at 37 °C. The blocking medium was discarded and 50 μL of cell suspension (300,000 cells/well) or medium (medium only wells) were added. The plates were placed back in the incubator (37 °C, 5% CO_2_) for at least one hour for the cells to settle down. Without disturbing the effector cells, an equal volume of medium (as the negative control), phytohemagglutinin (PHA) (Remel, Inc., Waltham, Massachusetts, USA), for a final concentration of 5 µg/mL, or antigen, for a final concentration of 5 μg/mL of each peptide, were added to the wells (the A2-CEF peptide pool was obtained from BioSynthesis, Lewisville, TX; individual 9mer peptides from JPT Peptide Technologies, Berlin, Germany; and TRP-2 peptide from PolyPeptide, San Diego, CA, USA); see Figure 1. Plates were wrapped in foil and placed at 37 °C in a 5% CO_2_ humidified incubator for at least 16 h. After incubation, the suspension was removed by flicking, and the plates were washed six times with PBS/0.05% Tween 20 (P/T20) on a plate washer (Biotek, Winooski, VT). An anti-IFNγ biotinylated antibody (Clone 7-B6-1, Mabtech, Cincinnati, OH) solution in PBS/0.5% BSA with a final concentration of 1 μg/mL was filtered with a 0.2-μm low protein-binding syringe filter (CORNING, Corning, NY) and added at 100 μL per well. The plates were wrapped in aluminum foil and returned to the incubator (37 °C, 5% CO_2_) for 2 h, followed by three washes with P/T20 and the addition of 100 μL/well of a streptavidin-HRP (Mabtech, Cincinnati, OH) solution prepared according to the manufacturer’s instructions. Plates were incubated at room temperature (RT) for 1 h, washed once with P/T20 and twice with PBS only. AEC (3-amino-9-ethylcarbazole) substrate (Vector Laboratories, Burlingame, CA) was added at 100 μL per well for 3 min, and spot development was stopped with three washes with dH_2_O (distillated water). The underdrain of the plate was removed to wash the back of the plate. The plate membranes were left to dry ON in the dark at RT in preparation for the evaluation. Figure 1 shows the different experimental conditions and peptides with their corresponding sequences tested in the study.” 

The authors would like to apologize for any inconvenience caused to the readers by these changes.
