# Supplementary material for: Improvement of IFNγ ELISPOT Performance Following Overnight Resting of Frozen PBMC Samples Confirmed Through Rigorous Statistical Analysis
Source: Cells. 2014 Dec 24;4(1):1–18. doi: 10.3390/cells4010001 (PMC4381205; doi:10.3390/cells4010001)
Supplement: Supplementary file 1 [file cells-04-00001-s001.zip › cells-67500-supplem-final/Supplementary Figure 1.pdf]

Supplementary Information

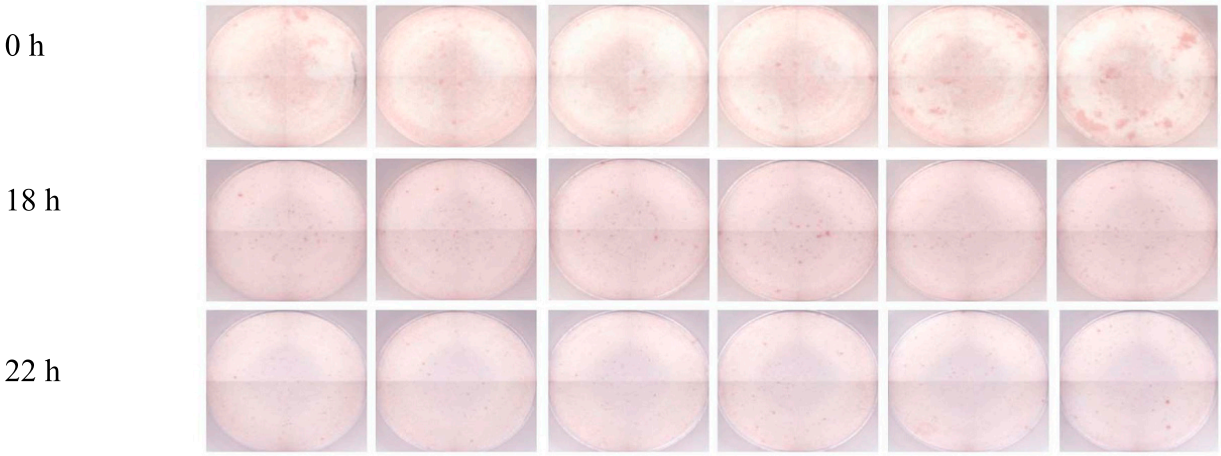

Figure S1. Artifacts in background wells can be cleaned by overnight PBMC resting. PBMC from H3 donor were no rested (0 h) or rested 18 h or 22 h previous to Elispot testing Shown pictures are from background control wells (no peptide added). Different donor vials were used for 0, 18 or 22 h resting.
