# Supplementary material for: Improvement of IFNγ ELISPOT Performance Following Overnight Resting of Frozen PBMC Samples Confirmed Through Rigorous Statistical Analysis
Source: Cells. 2014 Dec 24;4(1):1–18. doi: 10.3390/cells4010001 (PMC4381205; doi:10.3390/cells4010001)
Supplement: Supplementary file 1 [file cells-04-00001-s001.zip › cells-67500-supplem-final/Supplementary Table 3S.pdf]

MIANKA & MIATA Sub-Modules

#	Required	If available	Optional	Item
				Module 1 - Sample
				**Module 1A - Donor**
1.1	X			Essential donor info
				**Module 1B Source**
1.2	X			Source of cell material
1.3	X			Collection methodology
1.4		X		anti-coagulant, if available
1.5		X		Transportation/storage conditions for unprocessed samples, if available
1.6	X			Cell processing methodology
1.7		X		Median time and ranges from sample collection until end of cell processing, if available
1.8		☐		Cut-offs, if used
				**Module 1C - Cryopreservation and Storage**
1.9	X			Fresh or cryopreserved
				If cryopreserved
1.10	X			devices used
1.11	X			freezing process
1.12	X			medium used for freezing
1.13		X		Median time and temperature for each transportation and storage step, if available
1.14		☐		Cut-offs, if used
				**Module 1D - Cell Counting**
1.15	X			Median cell yield and viability (where available)
1.16		X		before freezing
1.17		X		after thawing
1.18		X		after overnight resting
1.19		☐		Cut-offs, if used
1.20	X			Cell counting methodology
1.21			☐	*Optional: Additional assessments*
				Module 2 - Assay
				**Module 2A - Medium/serum**
2.1	X			Medium/(serum) details
2.2	X			Pretesting info
				**Module 2B - Assay**
2.3		X		Treatment procedures of cells prior to assay, if applicable
2.4	☐			Sufficient assay details
				**Module 2C - Controls**
2.5	X			Internal assay controls
2.6		☐		Acceptance criteria, if available
2.7		☐		External reference samples, if used
2.8		☐		Assay acceptance criteria, if available
				Module 3 - Data Acquisition
				**Module 3A - Equipment and software**
3.1	X			Equipment and software version
3.2		X		Basic equipment settings, if available
				**Module 3B - Acquisition Strategy and Gating**
3.3	X			Detailed gating strategy or strategy for establishing spot detection parameters
3.4	X			Representative data set
3.5		X		Mean,median, ranges of event counts for relevant populations, if available
3.6			☐	*Optional: Unusual strategies explained*
3.7			X	*Optional: Review of raw data*
				Module 4 - Results
				**Module 4A - Raw data**
4.1		X		Background and ag-specific reactivity levels, if available
4.2		☐		Cut-off specifications and # of tests OOS, if available
4.3	X			Accessibility of raw data addressed?
				**Module 4B - Response determination**
4.4	X			Definition of positive reactivity (above background) including tests applied
4.5		X		Parameters, software and version used for response determination, if applicable
4.6	X			Response definition predefined or post-hoc?
4.7		☐		Definition of response induced by treatment, if applicable
4.8		☐		Any data excluded and why, if applicable?
4.9			X	*Optional: Why test was used*
				Module 5
				**Module 5A - General Lab Operation**
5.1	X			Guidance of lab operations
5.2		☐		Laboratory accreditions and certifications, if available
5.3			☐	*Optional: Details on audits*
				**Module 5B - Standardization**
5.4	X			Status of protocols
				**Module 5C - Qualification/Validation**
5.5	X			Status of assays
5.6			☐	*Optional: Specific performance criteria*
